# The effects of amino acid substitution of spike protein and genomic recombination on the evolution of SARS-CoV-2

**DOI:** 10.3389/fmicb.2023.1228128

**Published:** 2023-07-25

**Authors:** Letian Fang, Jie Xu, Yue Zhao, Junyan Fan, Jiaying Shen, Wenbin Liu, Guangwen Cao

**Affiliations:** ^1^Key Laboratory of Biological Defense, Ministry of Education, Shanghai, China; ^2^Shanghai Key Laboratory of Medical Bioprotection, Shanghai, China; ^3^Department of Epidemiology, Second Military Medical University, Shanghai, China; ^4^Department of Foreign Languages, International Exchange Center for Military Medicine, Second Military Medical University, Shanghai, China; ^5^School of Medicine, Tongji University, Shanghai, China

**Keywords:** SARS-CoV-2, evolution, Omicron, spike protein, amino acid substitution, recombination

## Abstract

Over three years’ pandemic of 2019 novel coronavirus disease (COVID-19), multiple variants and novel subvariants have emerged successively, outcompeted earlier variants and become predominant. The sequential emergence of variants reflects the evolutionary process of mutation-selection-adaption of severe acute respiratory syndrome coronavirus 2 (SARS-CoV-2). Amino acid substitution/insertion/deletion in the spike protein causes altered viral antigenicity, transmissibility, and pathogenicity of SARS-CoV-2. Early in the pandemic, D614G mutation conferred virus with advantages over previous variants and increased transmissibility, and it also laid a conservative background for subsequent substantial mutations. The role of genomic recombination in the evolution of SARS-CoV-2 raised increasing concern with the occurrence of novel recombinants such as Deltacron, XBB.1.5, XBB.1.9.1, and XBB.1.16 in the late phase of pandemic. Co-circulation of different variants and co-infection in immunocompromised patients accelerate the emergence of recombinants. Surveillance for SARS-CoV-2 genomic variations, particularly spike protein mutation and recombination, is essential to identify ongoing changes in the viral genome and antigenic epitopes and thus leads to the development of new vaccine strategies and interventions.

## 1. Introduction

Severe acute respiratory syndrome coronavirus 2 (SARS-CoV-2), a sister clade of SARS-CoV (Coronaviridae Study Group of the International Committee on Taxonomy of Viruses et al., 2020), has posed a global public health threat since its initial outbreak in December 2019 (Hu et al., 2020). On May 5, 2023, the World Health Organization (WHO) declared the end of the 2019 novel coronavirus disease (COVID-19) pandemic as a Public Health Emergency of International Concern. At that time, WHO reported a total of 765,222,932 cases and 6,921,614 deaths worldwide.^1^ Consistent with other coronaviruses, the genome of SARS-CoV-2 is a single-stranded positive-sense RNA of approximately 30,000 nucleotides, with replication mediated by RNA-dependent RNA polymerase (RdRP) (Vkovski et al., 2020; Li et al., 2020b). The 5’-terminus of the SARS-CoV-2 genome contains two open reading frames (ORFs), while the 3’-terminus contains four major structural proteins coding-gene in the following order: spike protein, envelope protein, membrane protein, and nucleocapsid protein (Bai et al., 2021). Despite the presence of error-correction enzymes, which contribute to a relatively high replication fidelity compared to other RNA viruses, SARS-CoV-2 still undergoes significant mutations (Robson et al., 2020; Domingo et al., 2021; Perales, 2021). The nucleotide mutation rates of SARS-CoV-2 are estimated to be 6.677 × 10^–4^ and 8.066 × 10^–4^ substitutions per year for the whole genome and spike protein, respectively (Wang S. et al., 2021).

Amino acid mutations in the spike protein play a crucial role in the evolution of SARS-CoV-2. The spike protein, which forms a trimeric fusion protein on the surface of the coronavirus, exhibits a crown-like appearance and serves as an ideal target for inducing neutralizing antibodies and protective immunity (Kang et al., 2021; Tian et al., 2021). The spike protein is composed of S1 and S2 subunits, and the Receptor Binding Domain (RBD) in the spike interacts with the human receptor angiotensin-converting enzyme 2 (ACE2) receptor when activated to allow the virus to entry into cells (Conceicao et al., 2020; Hoffmann et al., 2020b; Zhang et al., 2021a). Mutations in the spike protein, particularly in the RBD, have led to alterations in spike-ACE2 recognition, resulting in viral immune escape and the failure of neutralizing antibodies (Magazine et al., 2022; Chen et al., 2023). Spike proteins are classified as open and closed forms according to the up and down conformations of the RBD, and mutations in the spike may change the RBD conformation (Walls et al., 2020; Wrapp et al., 2020). The D614G mutation, which represents the substitution of amino acid D (Asp) by G (Gly), is conservative across all major variants (Wassenaar et al., 2022) and predominant in the spike protein during the early stage of pandemic (Chang et al., 2020). The D614G mutation has been shown to enhance furin proteolysis capacity by 50 times (Gobeil et al., 2021). Notably, the Omicron variant harbors more than 60 substitutions, deletions, and insertions, of which 15 rare mutations are found in the spike (He et al., 2021; Ma et al., 2022b). The spike protein of Omicron predominantly adopts closed conformations (Calvaresi et al., 2023), potentially leading to the failure of nearly all anti-spike monoclonal antibodies (Focosi and Casadevall, 2022; Turelli et al., 2022).

In addition to point mutations in the spike protein, viral genomic recombination is common among coronaviruses (Yewdell, 2021), especially during the late pandemic phase when different variants co-circulate. According to the US Centers for Disease Control and Prevention (CDC), the most prevalent circulating strains in the US as of May 13, 2023, were XBB.1.5 (61.5%), XBB.1.9.1 (10.0%), and XBB.1.16 (9.4%) (Ma et al., 2023). The frequent occurrence of recombination makes it challenging to predict the effectiveness of vaccines targeting the spike protein, and recombination may confer altered transmissibility, virulence, and immune escape properties to the virus (Focosi and Maggi, 2022; Carabelli et al., 2023).

**Figure 1:**
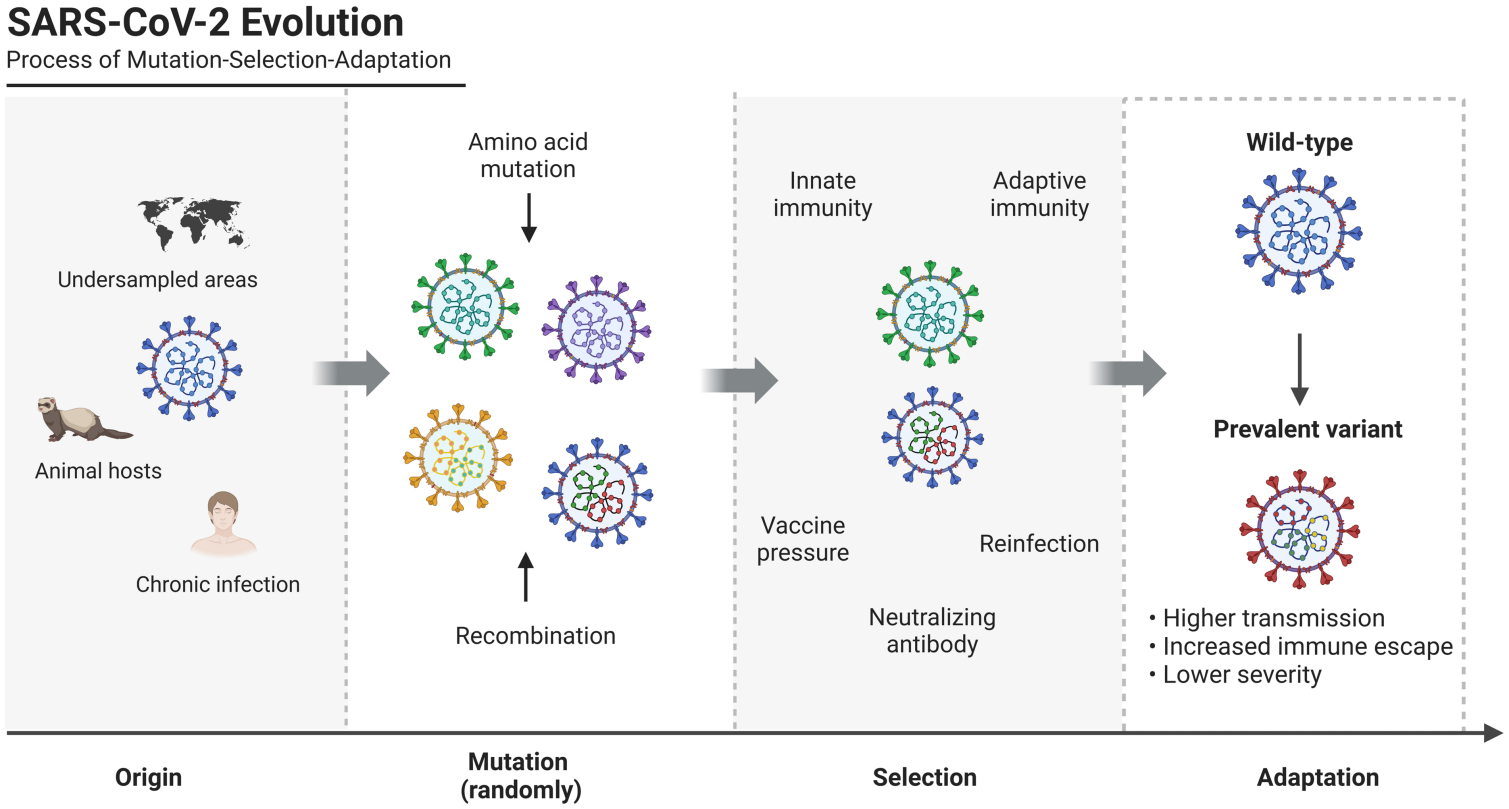
Process of mutation-selection-adaptation in SARS-CoV-2 evolution.

The evolution of SARS-CoV-2 within the population follows the mutation-selection-adaptation theory of Darwinian evolution (Goldman, 2021; Figure 1). In this context of hypermutation, both innate and adaptive host immune responses drive mutation selection (Thorne et al., 2021), as we have previously discussed (Shen et al., 2023). The virus evolves to adapt to external selection pressures, and antigenic drift occurs as mutations gradually accumulate, affecting the virus’s immunogenicity (Bano et al., 2021; Shapira et al., 2023). Antigenic drift facilitates viral evasion from host immune response, particularly by affecting antibody neutralization, resulting in viral resistance to previous infection and vaccination (Zhang et al., 2022; Cao et al., 2022c; Planas et al., 2023; Qu et al., 2023). The evolutionary trend tends to lower the pathogenicity but increase the transmissibility of variants, resulting in long-term retention of virus in human hosts (Magiorkinis, 2023). In this review, we provide an overview of SARS-CoV-2, summarize the characteristic amino acid mutations in the spike protein, particularly in novel variants, discuss recent recombination events, and propose future perspectives to guide viral evolution and intervention strategies.

## 2. An overview of SARS-CoV-2

### 2.1. Nomenclature and timeline of SARS-CoV-2

Several nomenclatures have been introduced for SARS-CoV-2 according to genetic relatedness of the sequences, including GISAID,^2^ Year-Letter (NextStrain) nomenclature,^3^ and Phylogenetic Assignment of Named Global Outbreak LINeages (Pango lineage) (Rambaut et al., 2020). The GISAID nomenclature system is based on marker mutations within the eight high-level phylogenetic groups, from the early split of S and L, to the further evolution of L into V and G, and later G into GH, GR and GV, and more recently GR into GRY. The Year-Letter nomenclature consists of the year when the clade emerged and a capital letter starting with A for each year, including 19A, 19B, 20A, 20B, 20C, and 20I. The Pango lineage uses an alphabetical prefix and a numerical suffix to identify descendants^4^ and contains phylogenetic, genetic, and epidemiological information. The first letter represents the lineage label of the variant, with the order from A to Z, then AA to AZ, BA to BZ, etc. The subsequent numbers separated by periods indicate the branches of lineages. When a branch has three more numeric suffixes, a new letter will be used as the lineage label in alphabetical order. For example, C.1 is the branch of B.1.1.1 (O’Toole et al., 2022). The recombinant variants are named in a uniform nomenclature beginning with “X.”

**Figure 2:**
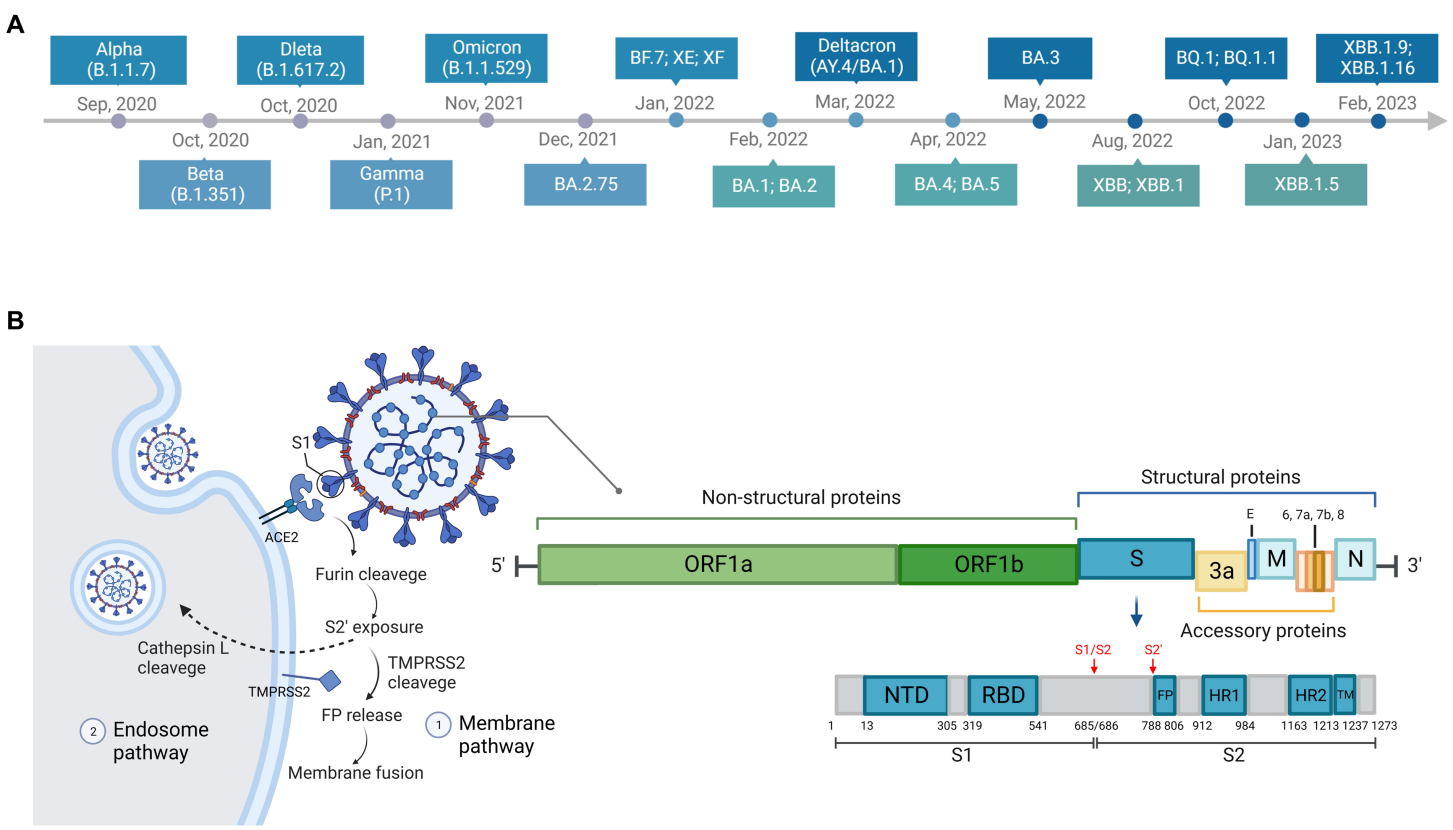
Timeline, structure, and entry pathways of SARS-CoV-2. **(A)** The chronological order of the emergence of major SARS-CoV-2 variants. **(B)** There are two pathways for SARS-CoV-2 entering cells: endosome pathway and membrane pathway. ACE2, angiotensin-converting enzyme 2; TMPRSS2, transmembrane protease serine protease 2; S1, subunit 1 of the spike protein; FP, fusion peptide, responsible for membrane fusion; S1/S2, furin cleavage site between S1 and S2 subunit of the spike protein; S2’, another proteolytic site in the subunit 2 of the spike protein.

To promote surveillance and research, WHO categorized SARS-CoV-2 variants as three specific classes: variants of concern (VOC), variants of interest (VOI), and variants under monitoring (VUMs).^5^ VOCs are variants of high mutation and transmission rate. To date, Alpha, Beta, Gamma, Delta, and Omicron are known emerged VOCs and have become dominant in turn globally or regionally. The Alpha variant (B.1.1.7) was discovered in the UK in September 2020 (du Plessis et al., 2021; Galloway et al., 2021). It was proven to be highly transmissible and infectious, and became prevalent a few months later (Davies et al., 2021; Volz et al., 2021). The Beta variant (B.1.351) was first reported in South Africa in October 2020 (Tegally et al., 2021), and the Gamma variant (P.1) was first identified in travelers from Brazil in January 2021 (Fujino et al., 2021). The Delta variant (B.1.617.2) was isolated in India (Mlcochova et al., 2021) and quickly became the most prevalent variant worldwide in June 2021 (Mahase, 2021). The Omicron variant (B.1.1.529/BA sublineages) was first discovered in Botswana, South Africa in November 2021, and outcompeted other VOCs rapidly upon its emergence (He et al., 2021). Five major sublineages of Omicron, BA.1, BA.2, BA.3, BA.4, and BA.5, have been identified so far (Tegally et al., 2022). Most recently, a series of novel Omicron subvariants have emerged, such as BA.2.75 (Saito et al., 2022), BF.7 (Scarpa et al., 2023a), Deltacron (Kreier, 2022), XE (Rahimi and Bezmin Abadi, 2022b), XF (Chakraborty et al., 2022), BQ.1 (Wang et al., 2022b), BQ.1.1 (Wang et al., 2022b), XBB (Imai et al., 2023), XBB.1 (Arora et al., 2023), XBB.1.5 (Tamura et al., 2022), XBB.1.16 (Harris, 2023), and they have raised increasing concern. The timeline of emergence of variants is illustrated in Figure 2A.

### 2.2. Entry pathways of SARS-CoV-2 and hypotheses for VOCs

Two described entry pathways of SARS-COV-2 through the cell membrane or through endosomes (Figure 2B) have been reviewed in detail previously (Shang et al., 2020; Hoffmann et al., 2020b; Rahbar Saadat et al., 2021; Jackson et al., 2021b; Lim, 2023). The two entry pathways differ because S2’ cleavage occurs either at plasma membrane by the transmembrane protease serine protease 2 (TMPRSS2) [such as in the nasal epithelial cells, lungs, and bronchial branches where TMPRSS2 is highly co-expressed with ACE2 (Lukassen et al., 2020; Sungnak et al., 2020)] or within the cell by endolyosomal cathepsins such as Cathepsin L (Bestle et al., 2020; Shang et al., 2020). The proteolytic site between the S1 and S2 subunit of the spike protein, also known as furin cleavage site (FCS), is cleaved by a host protease furin (Lavie et al., 2022). This process of cleavage is essential to the entry pathway and membrane fusion (Bestle et al., 2020; Hossain et al., 2021; Johnson et al., 2021; Peacock et al., 2021; Lavie et al., 2022). Optimization of FCS has been shown to facilitate cell–cell fusion to improve the infectivity (Hoffmann et al., 2020a), increase the transmissibility (Peacock et al., 2021), and promote pathogenesis (Johnson et al., 2021).

Multiple hypotheses have been proposed to explain the origin of VOCs (Mallapaty, 2022), such as (1) circulation in geographically sequencing limited areas; (2) circulation within animal hosts then spillover to humans; and (3) evolution in immunosuppressed chronic infection hosts. In some regions, the limited capacity for genomic sequencing has resulted in a lack of testing for asymptomatic patients. It has been observed that asymptomatic carriers exhibit higher levels of antiviral immunity and lower levels of inflammation compared to symptomatic individuals (Yang et al., 2020b; Le Bert et al., 2021; Ma et al., 2022a). This immunological profile may create an environment conducive to viral evolution under immune pressure. There is evidence supporting the hypothesis of an animal host origin, with white-tailed deer (Hale et al., 2022; Marques et al., 2022) and farmed mink (Koopmans, 2021; Lu et al., 2021) identified as stable animal reservoirs for SARS-CoV-2. These variants have the potential to infect animals and accumulate mutations within animal reservoirs. Subsequently, the virus may undergo further evolution, giving rise to new subvariants that can then spillover to humans. The hypothesis of chronic infection in immunodeficient hosts is widely accepted in many scenarios. Chronic infection in such individuals is associated with ACE2 affinity, immune evasion, and optimization of viral packaging (Choi et al., 2020; Kemp et al., 2021; Harari et al., 2022; Wilkinson et al., 2022). This process drives the mutation profiles of the virus and enhances its fitness (Ghafari et al., 2022; Hill et al., 2022). Extensive immune escape has been observed in SARS-CoV-2 infections in immunocompromised hosts, such as patients with advanced HIV disease (Cele et al., 2022).

## 3. Spike protein mutations produce antigenic drift

Mutation profiles of the variants of concern (VOCs) exhibit certain overlapping patterns, while also assuming distinct roles in the process of viral evolution, thereby suggesting an underlying evolutionary resemblance among these variants. Notably, a common early substitution mutation, namely D614G, is shared by all five VOCs, which has been shown to significantly augment the binding affinity of the viral spike protein to the ACE2 receptor, consequently amplifying viral pathogenicity (Alkhatib et al., 2021; Wang P. et al., 2021; Zhang et al., 2021b; Venkatakrishnan et al., 2022). Moreover, the substitution P681H has been identified in Alpha (Lubinski et al., 2022), Gamma (Fujino et al., 2021), and Omicron (Tian et al., 2022), and has been demonstrated to enhance viral cell entry. Conversely, the substitution P681R, occurring at the same position, has been observed to augment the replication capacity and pathogenicity of the Delta variant (Mlcochova et al., 2021; Saito et al., 2021; Liu et al., 2022). These mutations accumulate in a stepwise manner, progressively modifying the antigenic epitope of the virus, ultimately leading to a transition from “genetic drift” to antigenic drift.

### 3.1. Spike mutations in current VOCs

For variant Alpha (B.1.1.7), of eight mutations in the spike protein, D614G, Del H69/V70 (Del H69/V70 represents amino acid deletion mutation in the site 69 and 70 of the spike protein), N501Y, and P681H are most meaningful (Wang P. et al., 2021). The D614G mutation has been found to confer a fitness advantage by promoting efficient replication in primary airway cells, thereby increasing virulence and transmission (Hou et al., 2020; Korber et al., 2020; Ozono et al., 2021; Zhou et al., 2021). It also leads to alterations in spike conformation and enhanced FCS cleavage (Zhang et al., 2020) and leads to alterations in spike conformation and enhanced FCS cleavage (Gobeil et al., 2021; Nguyen et al., 2021). However, it has also been observed that the D614G mutation renders the virus more susceptible to monoclonal antibodies by increasing epitope exposure, suggesting that it does not impede the effectiveness of vaccines (Weissman et al., 2020), indicating it does not impede vaccine effect (Hou et al., 2020; Weissman et al., 2020; Yurkovetskiy et al., 2020; Ozono et al., 2021). Del H69/V70 is associated with diagnostic test failure for probes targeting spike proteins, known as spike gene targeting failure (SGTF) (Bal et al., 2021). SGTF has been utilized as a reliable proxy for monitoring the prevalence of the B.1.1.7 variant (Bal et al., 2021; Borges et al., 2021; Kidd et al., 2021). N501Y has been shown to enhance the binding of the spike protein to human ACE2 receptors, potentially expanding the host range of SARS-CoV-2 (Starr et al., 2020; Chan et al., 2021; Zahradník et al., 2021; Wang et al., 2022c). P681H, which is located adjacent to the FCS, has been found to enhance the efficiency of FCS cleavage during virus entry into cells and contributes to Alpha’s resistance to type I interferons (Lubinski et al., 2022; Lista et al., 2022).

For Beta variant (B.1.351), the combination of E484K and N501Y mutations has a synergistic effect in enhancing the affinity of the spike protein for human ACE2 receptors (Starr et al., 2020; Zahradník et al., 2021). Mutations Del 242–244, K417N, E484K, and N501Y have been shown to confer significant resistance to infection or vaccine-induced neutralizing antibodies (Garcia-Beltran et al., 2021; Hu et al., 2021; Tao et al., 2021; Wibmer et al., 2021; Wang P. et al., 2021).

The Gamma variant (P.1) carries 12 mutations in the spike protein, including K417T, N501Y, and E484K (Faria et al., 2021). These three mutations collectively enhance the affinity of the spike protein for ACE2 receptors, thereby increasing the transmissibility of the Gamma variant. E484K is also associated with reduced neutralization by antibodies (Faria et al., 2021). E484K is also associated with reduced neutralization by antibodies (Cele et al., 2021; Greaney et al., 2021; Wibmer et al., 2021).

The Delta variant (B.1.617.2) harbors several mutations previously reported in other VOCs, including L452R, T478K, E484Q, D614G, and P681R in the spike protein (Liu et al., 2022). These mutations partly explain the rapid global spread of the Delta variant upon its emergence. The L452R mutation has been found to increase infectivity, modestly reduce susceptibility to neutralizing antibodies, and enhance viral fusogenicity, thereby promoting virus replication (Motozono et al., 2021). E484Q exhibits similar reduced sensitivity to vaccine-induced neutralizing antibodies as L452R, but lacks synergistic effects when taken together (Motozono et al., 2021). Similar to P681H in Alpha, P681R in Delta increases FCS cleavage, resulting in enhanced transmissibility (Mlcochova et al., 2021; Saito et al., 2021; Wibmer et al., 2021). Studies have revealed that spike of Delta is more stable and binds with higher affinity to ACE2 than the spike of the wild-type (Gomari et al., 2023).

**Figure 3:**
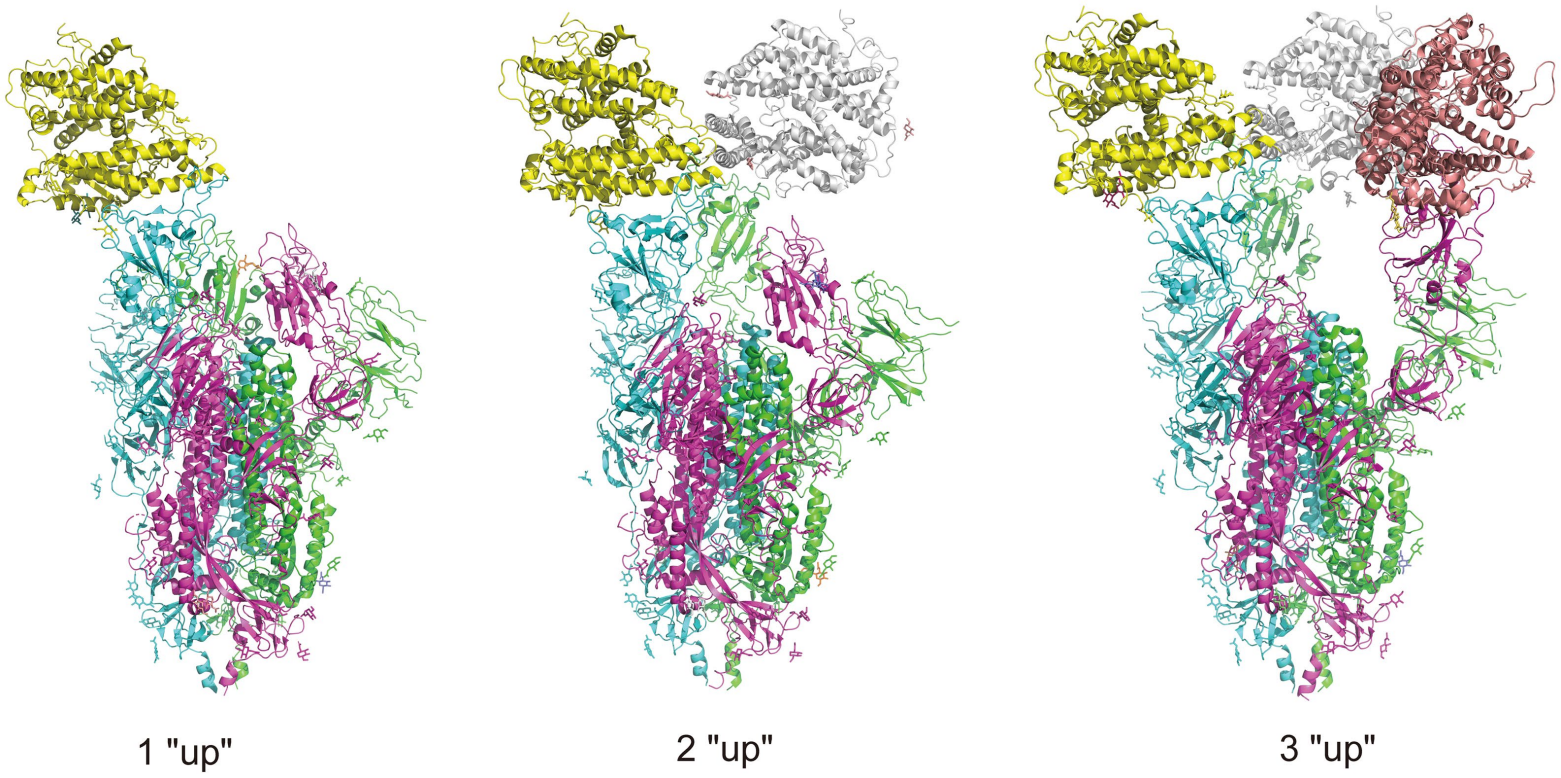
Illustration of RBD conformation of spike protein complexed with ACE2 receptors. There are two RBD conformations: “up” and “down,” and when the RBD is in “up” conformation, the spike protein is open to the ACE2 receptor. The trimeric spike protein is indicated by chain in three colors, purple, green, and blue, and three ACE2 receptors are indicated in yellow, gray, and pink. The complexes are obtained from RCSB.org (7KNE, 7KNH, 7KNI for 1 “up,” 2 “up,” 3 “up,” respectively).

As discussed above, the evolution of SARS-CoV-2 of pre-Omicron variants has primarily centered around recurrent mutations in key residues of the spike protein, including D614, N501, P681, K417, and E484. However, with the emergence of the Omicron variant and its sublineages, the landscape has undergone a significant shift. The Omicron variant harbors over 30 spike mutations, with 15 of them occurring in the RBD (Kumar et al., 2021). Figure 3 illustrates the mutation profiles of VOCs. In general, Omicron exhibits several distinctive characteristics compared to previous VOCs, including enhanced transmissibility, reduced antibody neutralization capacity (resulting in lower vaccine effectiveness), altered tissue tropism, relatively lower pathogenicity, and an increased likelihood of reinfection.

The higher transmissibility may attribute to the altered viral affinity to ACE2 receptor. Multiple experimental observations have demonstrated that the binding affinity between the RBD of the spike protein and ACE2 is significantly higher for Omicron compared to wildtypes (Kumar et al., 2021; Abeywardhana et al., 2022; Cui et al., 2022; Hong et al., 2022). The mutations T478K, Q493R, Q498R, and N501Y collectively contribute to the increased binding affinity through electrostatic effects (Kumar et al., 2021; Abeywardhana et al., 2022). However, another study revealed that Omicron exhibits comparable binding affinity to ACE2 when compared to the wild type SARS-CoV-2 and weaker binding affinity than the Delta variant (Wu et al., 2022). This discrepancy may stem from differences in the surface plasmon resonance methodologies employed in the studies, necessitating further research. The sublineages of Omicron display variations in their ACE2 affinity, with BA.2 exhibiting the highest affinity, followed by BA.3, BA.1, BA.2.75, and BA.5 (Abeywardhana et al., 2022). Furthermore, Omicron variants exhibit reduced sensitivity to neutralizing antibodies induced by triple-dose inactivated vaccines (Ren et al., 2023). Reports indicate that the neutralizing activity against Omicron variants is lost in 90% of immunization serum samples and 43% of convalescent serum samples (Zhang et al., 2022). In contrast to pre-Omicron variants, which primarily exploit TMPRSS2 for cell entry (Hoffmann et al., 2020b), Omicron variants have a propensity for entering nose and throat cells that are deficient in TMPRSS2 via the cathepsin-mediated endosomal pathway (Hui et al., 2022; Meng et al., 2022; Willett et al., 2022; Zhao et al., 2022). This shift in cell entry tropism from the membrane pathway to the endosomal pathway reduces the capacity of Omicron to fuse infected cells and form syncytia, resulting in a lower pathogenicity (Meng et al., 2022; Willett et al., 2022). The Omicron’s propensity to infect upper respiratory tract restricts its clinical manifestation and lowers the disease severity. From a structural standpoint, compared with Delta, Omicron has an inconsistent distribution of electrostatic potential and a geometric reorganization in the FCS of the spike protein. This structural divergence contributes to Omicron’s reduced fusogenicity and consequently lower pathogenicity (Fantini et al., 2022). Moreover, the Omicron variant possesses an enhanced capacity for immune evasion, leading to reinfection of individuals (Chavda et al., 2022; Xia et al., 2022). For pre-Omicron variants, infection-induced protective immunity has limited efficacy against BA.4 and BA.5, but it demonstrates a strong effect in preventing reinfection of BA.1 and BA.2 (Altarawneh et al., 2022).

Notably, the combinatorial mutations in the spike protein appear to have a synergistic effect on the characteristics of Omicron, further complicating its mutation profile. Preliminary findings suggest that certain mutations in Omicron form three distinct clusters, wherein the mutations seem to work in concert to compensate for the detrimental effects of any individual mutation (Martin et al., 2022). Two mutations, N501Y and Q498R, collectively increase the affinity of a variant for the ACE2 receptor by nearly 20-fold (Bate et al., 2022).

### 3.2. Spike mutations in novel subvariants

#### 3.2.1. BA.2.75 (BM.1.1.1)

BA.2.75, a descendant from BA.2, was first detected in India and Singapore (Saito et al., 2022). Differing from BA.2, BA.2.75 carries 9 additional mutations in the spike protein (147E, W152R, F157L, I210V, G257S, D339H, G446S, N460K, and an R493Q reversion mutation) (Sheward et al., 2022; Kurhade et al., 2023; Qu et al., 2023). BA.2.75 exhibits enhanced resistance to neutralization compared to BA.2 but falls short of the BA.4/5 variant (Qu et al., 2022; Saito et al., 2022; Cao et al., 2022b; Wang L. et al., 2022). The G446S and N460K mutations are primarily responsible for the increased resistance of neutralizing antibodies against BA.2.75 (Qu et al., 2022; Wang et al., 2022a), while the R493Q mutation reduces neutralization resistance (Wang et al., 2022a). Furthermore, the spike protein of BA.2.75 demonstrates significantly higher affinity for ACE2 (Saito et al., 2022), and the N460K mutation, which enhances S processing, leads to increased cell–cell fusion of BA.2.75 compared to BA.2 (Qu et al., 2022).

#### 3.2.2. BA.4.6

BA.4.6, a sublineage of BA.4, carries two additional mutations in the spike protein (R346T and N658S) and was initially identified in the US and UK (Hachmann et al., 2022). This subvariant exhibits a notable ability to evade neutralizing antibodies induced by infection or vaccination, with titers lower than those of BA.5 by a factor of 2 to 2.7 (Hachmann et al., 2022; Wang et al., 2022b; Planas et al., 2023).

#### 3.2.3. BF.7

BF.7 variant (also known as BA.5.2.1.7) is a derivative of BA.5 and has gained attention since the beginning of 2022, particularly in Asia (Kelleni, 2023; Pan et al., 2023; Scarpa et al., 2023a). Compared to BA.5, BF.7 carries an additional R346T mutation in the RBD and shares an identical N-terminal domain (NTD) (Scarpa et al., 2023a). The R346T mutation has been associated with enhancing the virus’s ability to evade neutralizing antibodies generated by vaccines or previous infection (Akif et al., 2023). However, R346T does not greatly increase the affinity of BF.7 to ACE2 (Scarpa et al., 2023a). Although enhanced resistance to neutralization exists (Qu et al., 2023), BF.7 appears to be less virulent, with a low evolutionary rate of 5.62 × 10^–4^ substitutions/sites/years compared to other Omicron subvariants (Scarpa et al., 2023a).

#### 3.2.4. CH.1.1

CH.1.1, a descendant of BA.2.75, has rapidly emerged in the UK. Compared with BA.2.75, CH.1.1 owns additional 4 substitutions (R346T, K444T, L452R, and F486S) in the RBD of the spike protein (Uraki et al., 2023). CH.1.1 does not pose a significant threat to pandemic control. Antiviral drugs (remdesivir, molnupiravir, nirmatrelvir, and ensitrelvir) remain effective against CH.1.1, and an additional dose of bivalent mRNA vaccines may be beneficial in preventing CH.1.1 infection (Uraki et al., 2023).

#### 3.2.5. BQ.1 and BQ.1.1

BQ.1 and BQ.1.1 have evolved from BA.5 (Wang et al., 2022b). Compared with the progenitor BA.5, BQ.1 carries additional K444T and N460K mutations in the spike protein, while BQ.1.1 has an additional R346T mutation (Wang et al., 2022b). Strong resistance to neutralization is observed in the BQ.1 and BQ.1.1 subvariants, largely driven by the N460K mutation (Kurhade et al., 2023; Qu et al., 2023).

#### 3.2.6. XBB and XBB.1.5

XBB variant carries 9 additional changes in the RBD and 5 additional changes in the NTD compared to its progenitor BA.2 (Imai et al., 2023). The R346 position is a critical mutation site (harboring R346T/S/I) that leads to increased immune evasion by neutralizing antibodies (Cao et al., 2021). Similar to BQ.1 and BQ.1.1, the XBB lineage exhibits an exceptionally strong ability to evade antibodies (Arora et al., 2023). BQ and XBB subvariants have rendered all authorized antibodies ineffective, with titers against BQ and XBB significantly lower (Wang et al., 2022a; Chakraborty et al., 2023). A cohort study in Singapore revealed that protection against XBB reinfection was lower and weakened more rapidly compared to protection against BA.4 or BA.5 reinfection in previously vaccinated omicron-infected individuals (Tan et al., 2023), further indicating greater immune evasion in XBB.

XBB.1.5 has a substantial growth advantage over BQ.1.1 and XBB.1, becoming the predominant strain in the US by January 2023 (Tamura et al., 2022). XBB.1.5 is a recombinant of two descendants from BA.2, differing from XBB.1 by an additional F486P mutation in the spike protein (Tamura et al., 2022). Unlike the F486S mutation in XBB.1, which disrupts the local hydrophobic interaction of the spike with ACE2, F486P in XBB.1.5 restores this interaction (Yue et al., 2023). This mechanism enhances the affinity for ACE2 and suggests a higher growth advantage for XBB.1.5 compared to its progenitor XBB.1. F486P makes XBB.1.5 slightly less immune evasive but more infectious than its ancestor XBB.1, likely due to increased binding affinity to human ACE2 (Tamura et al., 2022; Mahase, 2023).

#### 3.2.7. XBB.1.16

XBB.1.16 is another XBB sublineage harboring the F486P substitution, outcompeting other variants in India by the end of March 2023 (Looi, 2023; Varghese et al., 2023). Compared to XBB.1.5, XBB.1.16 carries two additional substitutions, E180V in the NTD and T478R in the RBD, in the spike protein (Yamasoba et al., 2023). XBB.1.16 exhibits a greater growth advantage compared to XBB.1 and XBB.1.5, but its potential for immune evasion is similar to XBB.1 and XBB.1.5 (Yamasoba et al., 2023). Notably, XBB.1.16 and XBB.1.5 demonstrate similar characteristics in terms of cell line tropism, cell entry efficiency, and neutralization evasion (Nehlmeier et al., 2023).

### 3.3. Spike mutations in RBD conformation

**Figure 4:**
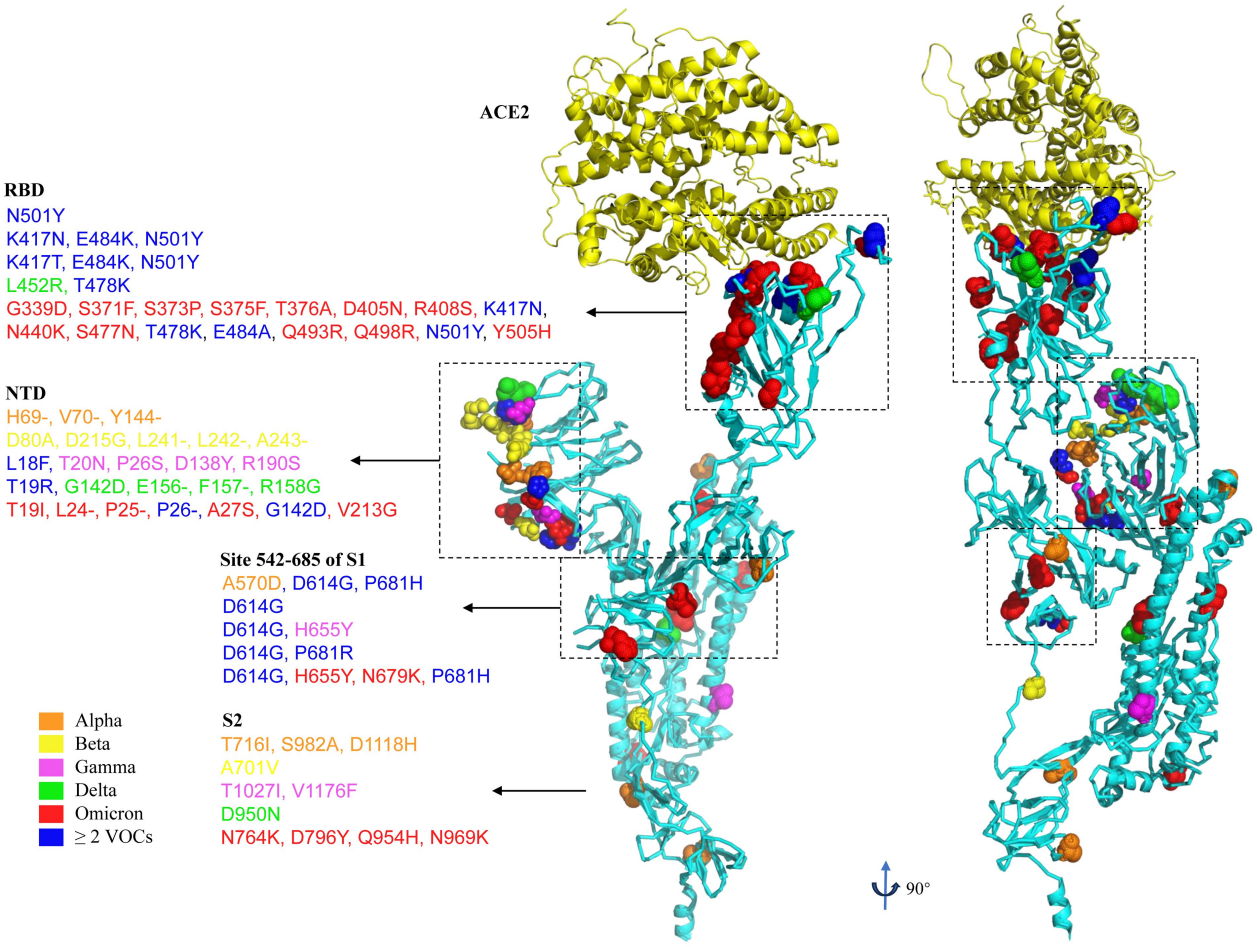
Representation of the SARS-CoV-2 spike protein, showing amino acid mutations in VOCs Alpha, Beta, Gamma, Delta and Omicron. Amino acid mutations are colored in orange, Alpha; yellow, Beta; purple, Gamma; green, Delta; red, Omicron; blue, ≥ 2 VOCs. The spike protein structure complexed with ACE2 receptor is obtained from RCSB.org (7KNE). The mutations of VOCs are based on the data from covariants (https://covariants.org, 20I for Alpha, 20H for Beta, 20 J for Gamma, 21A for Delta, and 21 L for Omicron).

SARS-CoV-2 infection is partially controlled by the conformation of the spike protein RBD. The RBD located in the S1 subunit of the extracellular domain of the spike is responsible for interacting with ACE2 receptors, and has been shown an important molecular determinant of the COVID-19 pandemic (Shang et al., 2020). The RBD exists in two different conformations: up for receptor binding and down for immune evasion. Accordingly, the spikes are also in open and closed conformations. Compared with the closed-form spike protein, an open-form with an up RBD conformation leads to infection more rapidly (Yin et al., 2022), and binding with antibodies more easily (Berger and Schaffitzel, 2020; Yin et al., 2022). Figure 4 illustrates the different up or down conformations of spike protein complexed with ACE2 receptors. In the early phase of the pandemic, the D614G substitution adjacent to the NTD subdomain leads to a more open and thus receptor-accessible conformations of the spike compared with the wild-type (Benton et al., 2021; Gobeil et al., 2021; Mansbach et al., 2021; Zhang et al., 2021a). The D614G substitution confers the virus an adaptation advantage and higher transmissibility, facilitating the acquisition of further mutations and forming the variants of concern (Korber et al., 2020; Zhang et al., 2020; Plante et al., 2021). It is shown that the conformations of Alpha, Beta and Delta spikes are predominantly open and that the binding of ACE2 increases membrane fusion (Calvaresi et al., 2023). In contrast, substitution of the Omicron spikes results in a predominantly closed conformation that may allow them to evade antibodies (Calvaresi et al., 2023). Other studies show that the mutations in the RBD of Omicron may promote the conformation to change from “down” to “up” and thus increase engagement of ACE2 (Hossen et al., 2022; Ye et al., 2022). This may due to the mutations that reduce the protein–protein interaction affinity of RBD with its neighboring domains (Singh et al., 2022).

Glycosylation is another way to affect the RBD conformation and thus change the spike open state. The SARS-CoV-2 spike gene encodes 22 N-linked glycan sequons per protomer and the trimeric spike protein displays 66 N-linked glycosylation sites. Glycosylated spike has a higher barrier to opening and also energetically favors the down state over the up state (Pang et al., 2022). Inhibition of protein N-glycosylation is shown to block SARS-CoV-2 infection (Casas-Sanchez et al., 2021). The glycosylation sites also have the effect of facilitating immune evasion by shielding specific epitopes from antibody neutralization (Watanabe et al., 2019). It is observed that proximal glycosylation sites (N165, N234, and N343) shield the receptor binding sites on the SARS-CoV-2 spike, especially when the RBD is in the “down” conformation (Watanabe et al., 2020). Sztain et al. (2021) revealed that N-glycan at position N343 facilitates RBD opening, and plays a gating role in the spike protein open state. Although the spike surface is substantially shielded by N-glycans, it presents regions that are vulnerable to neutralizing antibodies such as in the RBM, NTD, and S2 subunit (Chi et al., 2020; Tortorici et al., 2020; Cerutti et al., 2021). Mutations in the spike may affect glycosylation. For example, P681H and P681R were found in Alpha and Delta, respectively, and they decreased O-glycosylation which potentially increases furin cleavage and may influence viral infectivity (Zhang et al., 2021c).

## 4. Recombinant mutations complement variants with new properties

Recombination, a frequently observed evolutionary mechanism in coronaviruses, plays a significant role in the genetic diversity and evolution of these viruses. For example, lineage 5 of Middle East respiratory syndrome coronavirus (MERS-CoV), which caused the MERS-CoV outbreak in South Korea and mass infections in Saudi Arabia in 2015, is putatively a recombinant virus of groups 3 and 5 of clade B, or lineages 3 and 4 (Wang et al., 2015; Sabir et al., 2016). The measurement of recombination versus *de novo* mutation (R/M) provides insights into the relative impact of these two variations (Patiño-Galindo et al., 2021). In SARS-CoV-2, the R/M ratio is 0.00264 (Turakhia et al., 2022), while in MERS, it is estimated to be 0.25–0.31 (Patiño-Galindo et al., 2021), indicating a low level of recombinant mutations in the early stage of the SARS-CoV-2 pandemic. However, as co-infections and mutation accumulation increase within the population, recombination is expected to play a more prominent role in generating functional genetic diversity (Kim et al., 2020).

### 4.1. Co-circulation of variants provides basis for recombination

**Figure 5:**
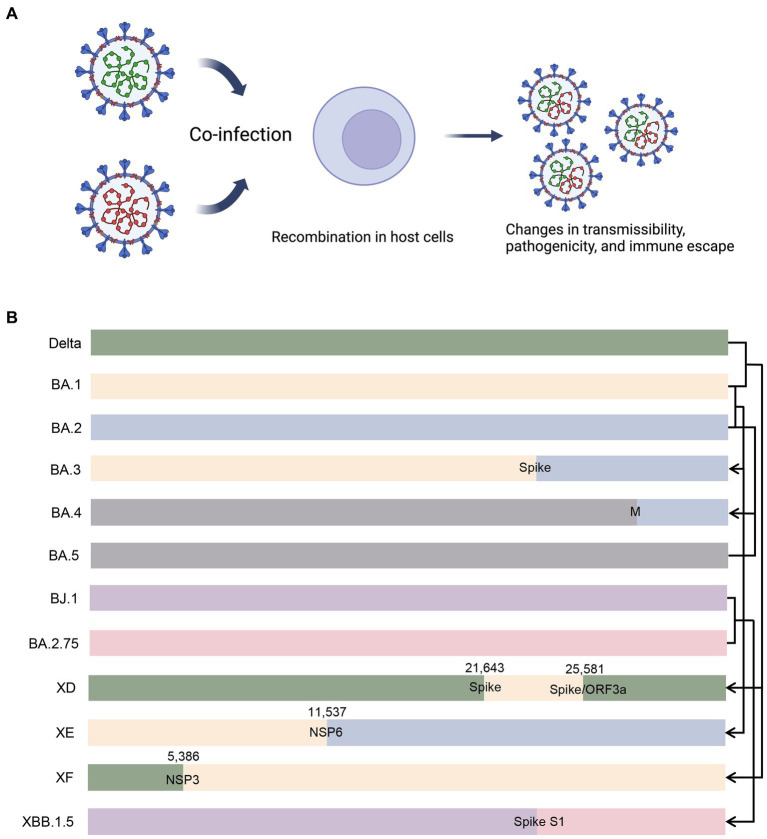
Illustration of recombination in co-infected cells and different recombination patterns. **(A)** When different variants co-infect an individual, there is possibility that recombinant variants emerge with altered properties. **(B)** BA.3 is putatively a recombinant of BA.1 and BA.2, and the breakpoint probably lies in the spike protein-coding gene. BA.4 is putatively a recombinant of BA.2 and BA.5, and the breakpoint probably lies in the M protein-coding gene. XD and XF are recombinants of Delta and BA.1, and the breakpoints lie in the spike protein-coding gene/ORF3a and NSP3 protein-coding gene, respectively. XE is a recombinant of BA.1 and BA.2, with breakpoint lying in the NSP6 protein-coding gene. XBB.1.5 is a recombinant od BJ.1 and BA.2.75, and the breakpoint probably lies in the S1 subunit of the spike protein-coding gene. M, membrane protein; ORF3a, open reading frame 3a; NSP, non-structural protein.

Recombination occurs when genetically distinct SARS-CoV-2 variants co-infect the same host during co-circulation (Figure 5A). This process leads to the emergence of recombinant viruses with new properties, such as increased transmissibility or virulence (Li et al., 2020a). Recombination occurs frequently in the later phase of pandemic (Varabyou et al., 2021). Turakhia et al. (2022) developed a method called Recombination Inference using Phylogenetic PLacEmentS (RIPPLES) to detect recombination in pandemic-scale phylogenies. By analyzing a 1.6 million sample tree, they identified 589 recombination events, indicating that approximately 2.7% of sequenced SARS-CoV-2 genomes have detectable recombinant ancestry (Turakhia et al., 2022). The distribution of recombination breakpoints across the SARS-CoV-2 genome is not uniform, with a higher incidence toward the 3’ end compared to the 5’ end, consistent with previous analyses in other human coronaviruses (Patiño-Galindo et al., 2021; Müller et al., 2022). Recombination events often lead to genetic alterations near the breakpoints, and the specific breakpoints vary across the genome (Bolze et al., 2022). For example, a recombinant virus containing genetic material from the Alpha (B.1.1.7) and Epsilon (B.1.429) variants was detected in New York, and recombinant mutations were found in the spike, nucleocapsid, and ORF8 coding regions (Wertheim et al., 2022). In the US, there have been nine reported recombination events between the Delta (AY.119.2) and Omicron (BA.1.1) variants, with the breakpoint located between the NTD and RBD of the spike protein (Lacek et al., 2022a). These recombinants can produce hybridized spike proteins containing characteristic amino acids from both Delta and Omicron (Lacek et al., 2022a). The co-circulation of different variants highlights the importance of ongoing genomic surveillance, with particular attention to recombinants (Jackson et al., 2021a). Figure 5B illustrates different patterns of recombination.

### 4.2. Co-infection in immunocompromised population accelerates recombination

Co-infection is common in the later phase of the pandemic. For example, a 17-year-old Portuguese female was reported to be co-infected with two SARS-CoV-2 lineages belonging to distinct clades, differing by six variants (Pedro et al., 2021). Similar co-infection events have been observed, such as B.1.1.28 co-infecting with either B.1.1.248 or B.1.91 lineages (da Silva Francisco et al., 2021), and GH co-infecting with GR clades (Samoilov et al., 2021). In the US, out of 29,719 SARS-CoV-2 positive samples sequenced from November 2021 to February 2022, 20 co-infections were identified (Lacek et al., 2022b). In Brazil, nine co-infection events (0.61%) were identified in the investigated samples from May 2020 to April 2021, although this data is likely an underestimation due to sample limitations. Recombination has been found to occur more frequently in immunodeficient individuals at high risk of severe COVID-19 (Perez-Florido et al., 2023). Immunodeficient individuals are considered incubators for punctuated evolutionary events, possibly due to their vulnerability to chronic and co-infections (Rockett et al., 2022). For instance, a recombinant variant of B.1.160 and Alpha was isolated from a patient with lymphoma who was chronically infected for 14 months. The patient was initially infected with B.1.160, followed by concurrent Alpha infection, and eventually, the recombinant variant emerged (Burel et al., 2022).

### 4.3. Intra-variant recombination in omicron major subvariants

Recombination occurs in five major sublineages of Omicron. BA.1, a descendent lineage of B.1.1, shows distinctly different phylogenetic as compared with other VOCs or VOIs. It has caused the fourth epidemic wave in South Africa (Araf et al., 2022; Lino et al., 2022; Saxena et al., 2022; Tian et al., 2022). The spike gene sequencing reveals that the BA.1 subvariant shares nine common amino acid mutations with most VOCs in the spiked proteins (three more than BA.2) (Araf et al., 2022; Ou et al., 2022; Tian et al., 2022), suggesting that Omicron may be derived from the recombinant origin of these VOCs. Three more Alpha-associated mutations (Del 69, Del 70, and Del Y144) were found in BA.1 rather than in BA.2, for BA.1 is phylogenetically closer to Alpha than the other variants (Kumar et al., 2021; Ou et al., 2022). Reverse mutations were also found in some dominant mutations (frequency > 95%) in BA.1 (Ou et al., 2022). Taken together, these support the role of Alpha in Omicron evolution.

Along with BA.1, BA.2 and BA.3 were also isolated in South Africa (Zhou et al., 2022). BA.2 has caused increased global infection, hospitalization, and mortality rate (Chen et al., 2022; Fonager et al., 2022; Rahimi and Bezmin Abadi, 2022a). BA.3 is likely a recombinant derivative of BA.1 and BA.2 due to BA.3 has similar genome in NTD region of the spike protein with BA.1 and BA.2 (Viana et al., 2022). A study revealed that BA.3 shared main mutations with BA.1 and BA.2, and BA.3 seemed to originate later (Wang C. et al., 2022), thus to some extent, corroborating the possibility of recombination.

BA.4 and BA.5 were afterwards identified as Omicron lineages in South Africa (Tegally et al., 2022). They were estimated to have originated in mid-December 2021 and early January 2022 (Viana et al., 2022). Their most recent common ancestor was estimated to have originated in mid-November 2021, coinciding with the emergence of BA.2 (Tegally et al., 2022). It deserves to note that BA.4 and BA.5 are close to BA.2 in genomes, and they both have similar spike proteins with BA.2 (Tegally et al., 2022). It is estimated that BA.4 and BA.5 are likely to evolve independently from the common ancestry of BA.2 subvariant (Wang C. et al., 2022). Compared with BA.2, BA.4 and BA.5 own extra mutations Del 69–70, L452R, F486V, and the wild-type amino acid at position Q493 (Ou et al., 2022). BA.4 and BA.5 share mutational profiles from 5’-UTR to envelope protein but differ distinctly from membrane protein to 3’-UTR (Tegally et al., 2022). This mutation pattern suggests that there exists a breakpoint within E and M, which is the possible evidence of recombinant event.

### 4.4. Inter-variant recombination between delta and omicron

Recombination events raised more concerns when Omicron quickly outcompeted Delta pandemic. Co-circulation of Delta and Omicron provided a grounded basis for recombinant variants. There is growing concern about the possibility that this recombination potential could eventually result in mutations that confer virus on enhanced transmissibility and immune escape properties.

On January 7, 2022, scientists detected a Delta and Omicron recombinant genome, and informally named it as “Deltacron” (Kreier, 2022). Nevertheless, it was later determined as a lab contamination (Kreier, 2022). On March 9, WHO declared the detection of such recombinants in different regions around the world and designated this Deltacron as a VUM (Farheen et al., 2022; Maulud et al., 2022). Generally, Deltacron is referred to as the AY.4/BA.1 recombinant, named XD, and consists of a full-length spike protein of Omicron and backbone of Delta (Mahase, 2022; Wang C. et al., 2022). According to Chinese Center for Disease Control and Prevention, of the 36 amino acid mutations found in the spike protein, 27 are present in BA.1 and 5 in AY.4, while 4 are present in both (Wang and Gao, 2022). Structural analysis of the Deltacron recombinant spike suggests its hybrid content leads to optimization of viral binding to the host cell membrane (Colson et al., 2022a,b). Consequently, this novel recombined virus causes increased disease transmission (Chakraborty et al., 2022; Hosch et al., 2022). The Deltacron recombinant also has the potential to escape neutralization by monoclonal antibody (Evans et al., 2022). Although Delta (AY.45) and BA.1 are sensitive to Sotrovimab neutralization, while an AY.45-BA.1 recombinant, with its breakpoint located adjacent to the Sotrovimab binding site, is resistant to its neutralization (Duerr et al., 2023). Deltacron shows higher transmissibility but lower clinical severity (Moisan et al., 2022). As recombination did not really emerge on a large scale and did not show its power until the appearance of Deltacron, the advent of Deltacron is regarded as a “gray rhino” event, rather than a “black swan” event.

Other than Deltacron (recombinant of AY.4 and BA.1, also known as XD), the UK Health security agency recognized two similar recombinants, XE and XF (Chakraborty et al., 2022). The XE recombinant contains genomic elements from Omicron BA.1 and BA.2 subvariants (Rahimi and Bezmin Abadi, 2022b). The breakpoint lies in the NSP6 protein-coding region of genome, with the 11,537 bp of the BA.1 and 11,537 bp of the BA.2 genomes before and after the break site (Chakraborty et al., 2022). XE appears to be roughly 10% more transmissible than its parent variant BA.2 (Basky and Vogel, 2022). The XF variant contains the genomes of NSP1 to NSP3 from the Delta variant; the breakpoint lies at site 5,386, and the rest genomes from Omicron BA.1 variant (Chakraborty et al., 2022).

XBB, nicknamed Gryphon, is the most recent recombinant. XBB is regarded as the first observed SARS-CoV-2 variant to increase its fitness through recombination rather than substitutions (Tamura et al., 2022). XBB derives from two BA.2 sublineages: BJ.1 (BA.2.10.1) and BM.1.1.1 (BA.2.75) (Arora et al., 2023; Scarpa et al., 2023b). XBB and its first descendant XBB.1 are both evolutionarily close to BA.2 genomes (Scarpa et al., 2023b), suggesting BA.2 acts as their progenitor. The breakpoint lies between position 22,901 and 22,939, a position in the middle of RBD (Scarpa et al., 2023b). The mutation profiles possibly altogether contribute to the greater immune invasion capabilities of XBB than do those of the earlier Omicron variants BA.2 (Imai et al., 2023). The pathogenicity of XBB.1 is comparable to or even lower than that of BA.2.75 (Tamura et al., 2022). Though XBB subvariants exhibit enhanced fusogenicity and substantial immune evasion in elderly population, but the fusion inhibitors EK1 and EK1C4 can potently block either XBB or XBB.1.5 spike protein mediated fusion and viral entry (Xia et al., 2023a).

### 4.5. Overall characteristics of emerging recombinants

As a whole, the novel recombinant subvariants demonstrate a higher transmission rate and relatively greater resistance to antibodies compared to earlier variants (Wang et al., 2022a; Brandolini et al., 2023; Faraone et al., 2023). In January 2023, there was a rapid increase in the prevalence of XBB.1.5 in the United States (Callaway, 2023). According to the World Health Organization (WHO), XBB.1.5 accounted for 23-86% of circulating variants throughout the country (XBB.1.5 Updated Risk Assessment, 24 February 2023).^6^ However, these recombinant variants do not significantly increase the severity of the disease or cause clinical exacerbation (Karyakarte et al., 2023). XBB.1.5 does not carry mutations associated with potential changes in pathogenicity, such as P681R (Mlcochova et al., 2021; Saito et al., 2021). It is important to note that most vaccines are developed based on the spike protein, and the emergence of recombinant variants may pose a risk of vaccine failure (Tamura et al., 2022). Therefore, it is crucial to consider potential new subvariants in the development of novel strategic vaccines.

## 5. Outlook for SARS-CoV-2 evolution and interventional strategies

Various factors drive the viral evolution (Moelling, 2021), including RNA polymerase exchanging accuracy for efficiency (Yewdell, 2021), the selective pressures exerted by host immune system (Milne et al., 2021; Thorne et al., 2021), chronic infection in other species then spillover to human (Lu et al., 2021; Hale et al., 2022; Marques et al., 2022), and prolonged co-infection in immunodeficient hosts (Ou et al., 2022; Rockett et al., 2022). These factors contribute to the mutation-selection-evolution process of SRAS-CoV-2 evolution. Continuous evolution of SARS-CoV-2 has led to rapid and simultaneous emergence of multiple variants that exhibit a growth advantage over previously circulating variants (Wolf et al., 2022). During the evolution of SARS-CoV-2, the spike gene is the only gene that undergo the strong positive selection, while other genes show only weak or temporary positive selection (Lu et al., 2023) Thus, spike mutations contribute highly in its evolution. The mutational process is dynamic, and the mutation spectrum of SARS-CoV-2 may tend to be more similar to that of other animal Sarbecoviruses (Bloom et al., 2022). Here we propose several interventional strategies.

1. Genomic surveillance of SARS-CoV-2, specifically in the spike gene and genomic recombination, is of utmost importance in recognizing its evolutionary trend. Efforts have been made to promote the genomic monitoring. Dadonaite et al. (2023) developed a novel deep mutational scanning (DMS) platform for mapping the effects of spike protein mutations on immune evasion and viral infectivity (Xia et al., 2023b). Saldivar-Espinoza et al. (2023) developed a SARS-CoV-2 Mutation Portal which provides access to a database of SARS-CoV-2 mutations. Sathyaseelan et al. (2023) developed a CoVe-tracker (SARS-CoV-2 evolution tracker)^7^ for quick surveillance of newly emerging mutations/variants/lineages to facilitate the understanding of viral evolution, transmission, and disease epidemiology. Huang et al. (2023) developed a genomic surveillance framework and a dynamic community-based variant dictionary tree, which enables early detection and continuous investigation of SARS-CoV-2 variants. Outbreak.info is a platform for scalable and dynamic surveillance of SARS-CoV-2 variants and mutations, and it relies on shared virus sequences from the GISAID Initiative (Gangavarapu et al., 2023; Tsueng et al., 2023).
2. As the recently expanding Omicron subvariants are capable of immune evasion from most of the existing neutralizing antibodies, it is imperative to explore broad-spectrum antivirals to combat the emerging variants. Resistance to monoclonal antibody neutralization is dominated by the action of epitope single amino acid substitutions in the spike protein (Cox et al., 2022). Currently, most therapeutic neutralizing antibodies and promising vaccine candidates are designed to target the RBD or use RBD as the sole antigen (Shi et al., 2020; Yang et al., 2020a, 2021; Dai et al., 2022; Han et al., 2022). A novel group of neutralizing antibodies and vaccines targeting S2 subunit of the spike [such as fusion peptide (FP), heptad repeats 1 and 2 (HR1-HR2), and stem helix (SH)] may become the next generation of therapeutic strategies. For example, COV44-62 and COV44-79 were identified as anti-FP antibodies and showed considerable neutralizing capacity (Dacon et al., 2022).
3. Strategies should be implemented to prevent long-term SARS-CoV-2 infection and to limit the spread of emerging, neutralization-resistant variants in immunocompromised patients (Gonzalez-Reiche et al., 2023). It is found that the evolutionary rate of SARS-CoV-2 in chronic infection individual is 2-fold higher than that around the globe (Chaguza et al., 2023). This persistent intrahost evolution may accelerate antigenic alteration and lead to the emergence of genetically distinct subvariants (Smith and Ashby, 2022; Ahmadi et al., 2023; Chaguza et al., 2023). Bendall et al. (2023) observed a tight transmission bottleneck that would limit the development of highly mutated VOCs in the transmission chain of acutely infected individuals, further suggesting that selection for long-term infection in immunocompromised patients may drive SARS-CoV-2 VOC evolution (Braun et al., 2021; Wilkinson et al., 2022). Surveillance by sequencing is recommended for (i) patients carried with SARS-CoV-2, (ii) patients suspected of reinfection, and (iii) patients who are immunocompromised (Landis et al., 2023).
4. Vaccination in large population acts as a valuable measure in decreasing the mortality. However, vaccination alone cannot slow the pace of viral evolution for immune evasion and therefore, vaccine protection against severe and fatal outcomes for COVID-19 patients may not be assured (Van Egeren et al., 2023). Current herd immunity and BA.5 vaccine boosters may not efficiently prevent the infection of Omicron convergent variants (Cao et al., 2022a). However, these may result from the decreased pathogenicity of SARS-CoV-2 via inducing the mutations. The vaccination against SARS-CoV-2 still efficiently decrease the case fatality rate (Wang C. et al., 2022).

## 6. Summary and conclusion

In the process of SARS-CoV-2 evolution, external and internal pressures drive the selection of randomly occurring mutations, with the retention of favorable mutations leading to adaptation. SARS-CoV-2 exhibits a trajectory of evolution characterized by increased transmissibility, reduced virulence, and enhanced immune escape, enabling its long-term persistence within the population. The mutation patterns observed in pre-Omicron variants primarily manifest at recurrent amino acid sites within the spike protein, affecting the RBD conformation and glycosylation sites, consequently altering antigenicity. However, the emergence of the Omicron introduced a multitude of novel mutations, resulting in a substantial increase in transmissibility and immune evasion. Remarkably, the severity and clinical manifestation of patients did not escalate further, mainly for Omicron’s tropism for the upper respiratory tract. These changes observed in Omicron are attributed to the ongoing viral evolution. The appearance of the recombinant variant XBB and its subsequent descendants since August 2022 likely stems from the co-circulation of multiple variants and co-infection in the immunocompromised patients during the later stage of the pandemic. Although novel recombinant variants such as XBB.1.5 and XBB.1.16 demonstrate a considerable transmission advantage and outcompete the predecessors, they do not exhibit a significant increase in disease severity and display relatively moderate antibody escape. Although SARS-CoV-2 is no longer regarded as a Public Health Emergency of International Concern, its evolution persists. We strongly recommend for enhanced surveillance of the viral genome, particularly in immunocompromised patients, the development of therapeutics targeting domains beyond the RBD, and the promotion of widespread vaccination.

## Author contributions

GC and LF: conceptualization. LF, JX, YZ, JF, and JS: data collection. LF, JX, and GC: writing—original draft preparation. LF, JX, GC, YZ, JF, WL, and JS: writing—review and editing. All authors have read and agreed to the published version of the manuscript.

## Funding

This work was funded by the National Natural Science Foundation of China (82041022) and Shanghai Commission of Science and Technology (20JC1410200 and 20431900404).

## Conflict of interest

The authors declare that the research was conducted in the absence of any commercial or financial relationships that could be construed as a potential conflict of interest.

## Publisher’s note

All claims expressed in this article are solely those of the authors and do not necessarily represent those of their affiliated organizations, or those of the publisher, the editors and the reviewers. Any product that may be evaluated in this article, or claim that may be made by its manufacturer, is not guaranteed or endorsed by the publisher.

## Footnotes

^1^ https://covid19.who.int/

^2^ https://www.gisaid.org/references/statements-clarifications/clade-and-lineage-nomenclature-aids-in-genomic-epidemiology-of-active-hcov-19-viruses/

^3^ https://nextstrain.org/ncov

^4^ https://www.pango.network/the-pango-nomenclaturesystem/statement-of-nomenclature-rules/

^5^ https://www.who.int/en/activities/tracking-SARS-CoV-2-variants/

^6^ https://www.who.int/

^7^ https://project.iith.ac.in/cove-tracker/
